# Regulation of the renal sympathetic nerves in heart failure

**DOI:** 10.3389/fphys.2015.00238

**Published:** 2015-08-25

**Authors:** Rohit Ramchandra, Carolyn J. Barrett

**Affiliations:** Department of Physiology, The University of AucklandAuckland, New Zealand

**Keywords:** heart failure, renal sympathetic nerve activity, central control

## Abstract

Heart failure (HF) is a serious debilitating condition with poor survival rates and an increasing level of prevalence. HF is associated with an increase in renal norepinephrine (NE) spillover, which is an independent predictor of mortality in HF patients. The excessive sympatho-excitation that is a hallmark of HF has long-term effects that contribute to disease progression. An increase in directly recorded renal sympathetic nerve activity (RSNA) has also been recorded in animal models of HF. This review will focus on the mechanisms controlling sympathetic nerve activity (SNA) to the kidney during normal conditions and alterations in these mechanisms during HF. In particular the roles of afferent reflexes and central mechanisms will be discussed.

## Introduction

Heart failure (HF) is a complex syndrome, arising secondary to a wide range of cardiac structural and functional abnormalities, with the manifestations being shortness of breath, fatigue, exercise intolerance, and oedema. Elevated sympathetic drive is well recognized to play a key role in the pathophysiology of HF, with increased sympathetic nerve activity (SNA) to the kidneys resulting in renal vasoconstriction, increased renal sodium retention and increased renin release, and consequently elevated angiotensin II and aldosterone levels (DiBona and Kopp, 1997). In turn, angiotensin II is known to drive adverse cardiac remodeling and deterioration in cardiac function via a number of different pathways, including inflammatory pathways and fibrosis (Zhao et al., 2014). While initially activation of renal sympathetic nerve activity (RSNA) may assist in maintaining cardiac output, in the long-term neurohumoral activation drives the fluid overload associated with HF, significantly impacting both morbidity and mortality.

The significance of the sympathetic activation in HF is highlighted by the successful use of ACE inhibitors and β-blockers in the clinical management of the syndrome (Yancy et al., 2013). β-Blockers are well established to reduce the rate of mortality (Esler and Kaye, 1998), however, even when the majority of patients are prescribed β-blockers, increased renal norepinephrine (NE) spillover is directly linked to mortality (Petersson et al., 2005). Kidney function is an important determinant of outcomes during HF, with glomerular filtration rate, being an independent predictor of mortality in HF (Hillege et al., 2006). Understanding the mechanisms regulating renal function, and the changes that occur during the development of HF is thus crucial in understanding the pathophysiology of HF. In this review we will review the evidence for an increase in RSNA in HF and focus on three key areas potentially involved in mediating the changes: the arterial baroreceptors, the peripheral chemoreceptors, and the central regulation of RSNA within the paraventricular nucleus of the hypothalamus. A more in depth discussion of additional afferent signals such as from cardiopulmonary volume receptors and the afferent kidney reflex in control of RSNA in HF is presented by Booth et al. in this same special edition.

## Resting levels of renal sympathetic nerve activity in HF

**Figure 1:**
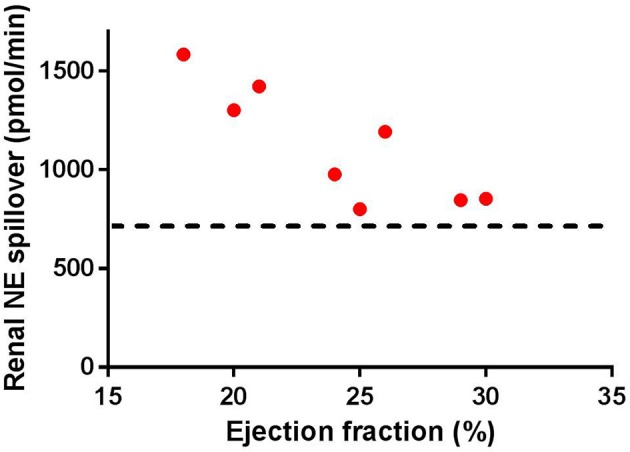
**Renal norepinephrine (NE) spillover increases as the ejection fraction decreases in separate groups of heart failure patients (individual numbers taken from Rundqvist et al., 1997; Brunner-La Rocca et al., 2001; Al-Hesayen and Parker, 2004, 2008; Petersson et al., 2005**. Note the straight line indicates the average baseline renal NE spillover in normal age-matched controls (taken from Rundqvist et al., 1997; Petersson et al., 2005).

Early studies in patients with severe HF have indicated that the spillover of NE from the kidney is increased (Hasking et al., 1986; Aggarwal et al., 2003). Figure 1 illustrates the progressive increase in spillover of NE from the kidney observed in clinical studies. As the figure indicates, as the progression of HF reaches ejection fractions of below 30%, there is a significant increase in renal spillover of NE. Rundqvist et al. (1997) indicate that at ejection fractions of 29 ± 7%, the renal spillover of NE is not significantly different to a normal matched cohort. However, once ejection fraction decreases below 25%, there is a significant increase in renal spillover of NE. Interestingly at ejection fractions of 29%, a three-fold increase in cardiac spillover of NE is observed indicating that the increase in cardiac spillover of NE occurs earlier than that in the kidney (Rundqvist et al., 1997).

An increase in spillover of NE from the kidney may be driven by changes in directly recorded SNA or may be secondary to changes in reuptake of NE at the synapse. Various animal models have been employed to study the role of the renal sympathetic nerves in HF. These include the myocardial infarction (MI) induced HF model in the rat and pacing induced HF in the rabbit, sheep, and the dog. The majority of studies have indicated an increase in directly recorded RSNA during HF with ejection fractions of 36–45%. The MI induced model of HF in rodents results in a significant increase in baseline resting levels of RSNA (DiBona et al., 1996, 1997). Similar to this, the pacing induced model of HF in rabbits is also associated with an increase in baseline levels of sympathetic drive to the kidney (Sun et al., 1999a; Liu et al., 2000). The finding of a significant increase in RSNA is not universal in all animal models of HF. Previous studies have found that baseline levels of RSNA are not increased in doxorubicin induced HF (Rossi et al., 2008) or in pacing induced HF in sheep (Ramchandra et al., 2009a). Given the progressive increase in renal spillover of NE as HF worsens, it is possible that these findings merely reflect models of HF which are not severe enough.

### Clinical relevance

It is interesting that animal models of HF that have indicated an increase in RSNA show increases at ejection fractions of 50% while clinically at the same stage of HF, there is no increase in renal spillover of NE. Considering this discrepancy, it is important to note that the majority of HF patients are on medications in particular ACE inhibitors and angiotensin II receptor blockers. Given that angiotensin II has been shown to facilitate release of NE from sympathetic nerve endings (Johnson et al., 1974; Burgdorf et al., 2003), it is tempting to speculate that any effects of RSNA in moderate stages of HF may be attenuated by these drugs although direct evidence of this is currently lacking. In addition, the re-uptake of NE at the synapse in the kidney is lower compared to the heart and this has meant that the role of reuptake mechanisms in modulating spillover of NE in the kidney has not been adequately explored.

## The role of afferent inputs in regulating RSNA

**Figure 2:**
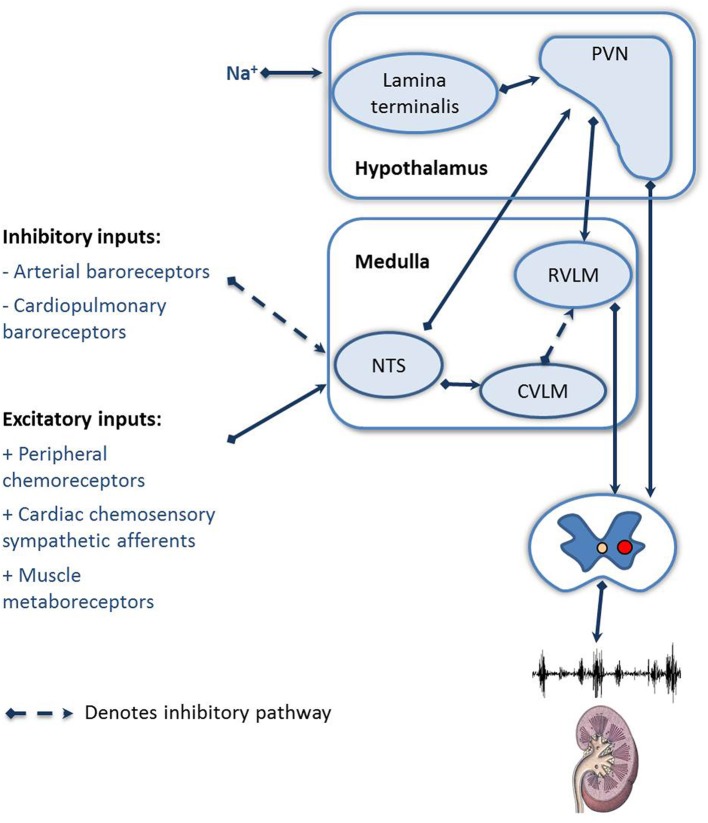
**Schematic showing the afferent reflexes which modulate renal sympathetic nerve activity during heart failure**. NTS, Nucleus of the solitary tract; RVLM, rostral ventral lateral medulla; CVLM, caudal ventral lateral medulla; PVN, paraventricular nucleus of the hypothalamus.

The regulation of RSNA is dependent on the integration of many afferent inputs, including the cardiopulmonary receptors, cardiac chemoreceptor afferents, and feedback from muscle receptors (Figure 2). In HF the general consensus is that there is a blunting of the inhibitory reflexes, including the arterial and cardiopulmonary baroreceptors and an increase in the sensitivity of the excitatory reflexes, including the peripheral chemoreceptors, cardiac chemosensory afferent reflexes, and muscle metaboreceptors (as reviewed by Floras, 2009; Zucker et al., 2012). Recently focus has been drawn to the role of the arterial baroreceptor reflex and the peripheral chemoreceptors, due to the potential to modulate these reflexes clinically. We will focus on these two afferent reflexes.

### Arterial baroreceptor reflex

Perhaps the most well recognized modulator of SNA is the arterial baroreceptor reflex. With receptors located primarily in the carotid sinus and aortic arch any increase in stretch of the arterial wall results in an increase in firing of afferent baroreceptor nerve activity. Stimulation of the arterial baroreceptors results in inhibition of SNA, playing an important role in buffering rapid changes in arterial pressure (Lohmeier and Iliescu, 2015). While initial studies suggested that the arterial baroreflex plays a minor role in chronic regulation, more recent studies indicate arterial baroreceptor reflexes are capable of regulating arterial pressure over much longer periods (Thrasher, 2005; Lohmeier et al., 2009). Sustained baroreflex-mediated inhibition of RSNA is observed in response to increases in arterial pressure induced by pressor doses of angiotensin II in both rats and rabbit models (Barrett et al., 2003, 2005; Yoshimoto et al., 2010), suggesting that the baroreflex is capable of influencing RSNA chronically. Whether the baroreflex plays a role in driving the changes in RSNA observed during HF remains controversial. Most reports have suggested that in HF arterial baroreflex control of RSNA is blunted, with a reduced sensitivity (DiBona et al., 1995; Murakami et al., 1997) and often also a reduced range of control (DiBona and Sawin, 1994; Liu et al., 1996; Zhang et al., 1999). As a consequence it is often argued that blunting of the arterial baroreflex may play a permissive role in HF, allowing the excitatory reflexes to predominate, resulting in an increase in RSNA (DiBona and Sawin, 1995). However, there is growing evidence that the arterial baroreflex regulation of RSNA in HF is more complicated than first presented. Recent research suggests that the degree of HF, female sex, and methods of normalizing RSNA can all influence the interpretation of data (Pinkham et al., 2015). Pinkham et al. showed that a blunting of arterial baroreflex regulation of RSNA was positively associated with deterioration in left ventricular function in males and ovariectomized females, but not ovary intact females, and only when the data was normalized (Pinkham et al., 2015). These findings fit with those of Ramchandra et al. (2009a) who have demonstrated that arterial baroreflex control of RSNA is unaltered in conscious female sheep with mild pacing induced HF. It should also be noted that elevations in plasma NE levels are still observed with the development of HF in dogs with baroreceptor denervation (Brändle et al., 1996). Thus, while the majority of studies suggest that at least in males with severe HF there may be an impaired ability of the baroreflex to inhibit RSNA, it is difficult to suggest that the arterial baroreceptor reflex is the primary driver for the increase in RSNA associated with HF. Furthermore, clinical studies suggest that whilst baroreflex control of heart rate (HR) is impaired in HF, there is little evidence to suggest that impairment of the baroreceptor reflex control of muscle SNA plays a role in driving the increase in muscle SNA (Floras, 2009).

### Peripheral chemoreflex

The carotid body (CB) is the most perfused organ per gram weight in the body [2000 ml min^−1^ (100 mg tissue)^−1^]. It receives its blood supply via an arterial branch arising from the internal or external carotid artery, which ensures that the CB responds acutely to changes in arterial oxygen levels and other contents (e.g., CO_2_, acidotic pH, hypoglycaemia, and hypoperfusion). Stimulation of the CB drives an increase in systemic sympathetic tone through direct signaling to the nucleus tractus solitarii and rostral ventrolateral medulla (Marshall, 1994). The CB chemoreflex not only serves as a protective mechanism during hypoxia (Prabhakar, 2013), but also contributes eupnoeic drive to breathing and control of blood flow during exercise (Dempsey, 2012). Activation of the CB by hypoxia or chemical stimuli increases respiratory drive and SNA to various vascular beds (Xing and Pilowsky, 2010), including the heart (Kollai et al., 1978).

More recently the role of the CB chemoreflex has been implicated in mediating the increased RSNA during HF (Li et al., 2005, 2006; Schultz and Li, 2007; Ding et al., 2011) and hypertension (McBryde et al., 2013; Paton et al., 2013; Moraes et al., 2015). In a rabbit model of pacing-induced HF, Schultz and colleagues have documented that maladaptive CB activity not only contributes to the enhanced RSNA response to hypoxia (Ding et al., 2011), but also provides a tonic excitatory influence to increase RSNA (Sun et al., 1999b). This enhanced responsiveness appears to be mediated at least in part by the CB chemoreflex, and depends on reduced CB blood flow, increased angiotensin II, and down-regulation of nitric oxide (NO) (Li et al., 2005, 2006; Ding et al., 2011). Importantly, inhalation of 100% oxygen decreased RSNA levels in pacing-induced HF animals to a greater extent than in normal animals (Sun et al., 1999b), suggesting a tonic excitatory input from this reflex. Interestingly, acute inhibition of the CB also reduces cardiac SNA (Xing et al., 2014) and consistent with this, denervation of the CB has led to an improvement in heart function (Del Rio et al., 2013) further strengthening the case for targeting the CB in HF.

#### Clinical relevance

In clinical studies, the issue is less clear with some reports finding that breathing 100% oxygen does not decrease muscle SNA in HF (van de Borne et al., 1996), whereas others have found reduced SNA suggesting a role for tonic chemoreflex activation (Despas et al., 2009; Franchitto et al., 2010). This discrepancy is probably due to the variety of methods used to assess peripheral chemoreflex sensitivity, and the patients selected for the studies. The discrepancy could also reflect differential control of SNA to the muscle and the kidney. Additionally and more importantly, only 40% of patients with HF indicate an augmented response to the chemoreflex (Chua et al., 1997). This was classified as an increase in ventilation rate during hypoxia induced using transient inhalations of 100% nitrogen. We can conclude that there is clearly a large percentage of the HF population where any putative increases in RSNA are driven by other mechanisms. Why some patients are sensitive to the chemoreflex but others not remains unclear. This may be related to anemia induced by HF since one previous study has shown that deactivation of chemoreceptors reduces muscle SNA in chronic HF patients with anemia but does not change levels of muscle SNA in chronic HF patients with no anemia (Franchitto et al., 2010). These subtleties of HF have not been adequately reproduced in animal models of HF and remain an important area where more basic research is required.

## Central regulators of RSNA

We will now shift focus from the afferent reflexes to central brain regions which integrate the input of these reflexes. In this context, sympathetic drive to various organs arises from sympathetic premotor neurons in the central nervous system. These include the “traditional” sympathetic premotor regions such as the rostral ventral lateral medulla, the rostral ventral medial medulla, the medullary raphe, the paraventricular nucleus of the hypothalamus (PVN), and the A5 noradrenergic cell group (Strack et al., 1989; Schramm et al., 1993) as well as recent additions including the locus coeruleus, lateral hypothalamus, and periaqueductal gray (Cano et al., 2004), Barrington's nucleus (Cano et al., 2000) and subcoeruleus nucleus (Cano et al., 2004). When it comes to ascertaining the central brain regions involved in regulating sympathetic drive to the kidney the majority of studies have focused on the PVN due to the important role this region plays in blood volume regulation. Anatomically the PVN includes vasopressin-producing magnocellular neurons and parvocellular neurons which project to both the spinal preganglionic sympathetic neurons and premotor sympathetic neurons in the brain stem (Shafton et al., 1998). Moreover, plasma and CSF [Na^+^] are monitored via sodium/osmo receptors in the lamina terminalis (McKinley et al., 2003) and studies have indicated an important pathway from the lamina terminalis to the PVN in mediating changes in RSNA (May et al., 2000; Shi et al., 2007).

### Role of the PVN in mediating increased RSNA during HF

Previous studies that have examined the role of the PVN in the baseline control of RSNA in normal animals have found variable effects. Microinjection of muscimol, a GABA receptor agonist into the PVN, results in increases (Badoer et al., 2002), decreases (Zhang et al., 2002; Akine et al., 2003), or no change in resting levels of RSNA (Ng et al., 2004; Stocker et al., 2004, 2005; Ramchandra et al., 2013). These different RSNA responses are likely to be due to the effects of anesthesia in previous studies, as anesthesia can significantly modify the responses to stimulation of the PVN (Kannan et al., 1989). These data suggest that in conscious animals, there is little contribution by the PVN to baseline levels of RSNA, although we cannot categorically rule out a tonic inhibitory role of the PVN in mediating baseline levels of RSNA (Zhang et al., 2002; Ramchandra et al., 2013).

In contrast to normal animals, the PVN appears to play an important role in mediating the high resting levels of RSNA observed in animal models of HF. Activation of the PVN in HF is indicated by the increased expression of *c-fos* as well as *Fos* related antigens in neurons in the PVN (Vahid-Ansari and Leenen, 1998; Patel et al., 2000). More importantly, studies in a rat model of HF indicate that activation of the PVN contributes to the increased levels of RSNA (Li and Patel, 2003; Zheng et al., 2009). Microinjection of muscimol into the PVN of rats with HF results in diminished depressor responses in MAP, HR, and RSNA (Patel, 2000; Zhang et al., 2002; Wang et al., 2009) suggesting a reduction in the GABAergic inhibitory input to the PVN may mediate the increase in baseline levels of RSNA (Carillo et al., 2012).

In terms of neuromodulators involved, studies have focused on the role of NO within the PVN. Reduced synthesis of NO, due to down regulation of the neuronal isoform of nitric oxide synthase (NOS), has been suggested as a cause of the centrally mediated sympathoexcitation in HF. In conscious normal rats, unilateral PVN microinjections of the NO donor, sodium nitroprusside (SNP) (0.5–1.0 M, about 13–26 μg), decreased MAP (Martins-Pinge et al., 2012). This is also associated with a decrease in RSNA in anesthetized rats (Zhang et al., 1997) indicating a sympathoinhibitory action of NO within the PVN. Although the inhibition of RSNA in these studies is likely a direct central effect of SNP, as the renal inhibition occurred in the presence of a fall in blood pressure, it is important to note that the intravenous dose of SNP required to produce large falls in blood pressure in rats is 5–10 μg. This raises the possibility that changes observed after SNP infusions or microinjections may result from supraphysiological increases in NO levels. In this regard, studies with inhibition of NOS are clearer to interpret. Acute inhibition of central NOS with the nonselective inhibitor *N*^ω^-nitro-l-arginine methyl ester (l-NAME), microinjected in the PVN increased baseline levels of RSNA (Zhang et al., 1997; Zhang and Patel, 1998). These findings suggest that NO within the PVN regulates baseline levels of RSNA in rodents however it is important to note that in conscious sheep and rabbits, this is not the case (Ng et al., 2004; Ramchandra et al., 2014).

Previous studies have indicated that neuronal NOS (nNOS) levels are decreased in HF, in particular, in neurons of the PVN (Patel et al., 1996a; Zhang et al., 1998; Ramchandra et al., 2014). The functional importance of this as a cause of the increased RSNA in HF is shown by the beneficial effects of nNOS gene transfer into the PVN (Zheng et al., 2011). Administration of a recombinant adenovirus that overexpressed nNOS resulted in an attenuated increase in HR and RSNA due to microinjection of N-methylD-aspartic acid into the PVN (Zheng et al., 2011). These studies indicate that during HF the PVN is at least partly responsible for the increase in baseline levels of RSNA.

Studies have also examined the role of the PVN in modulating the reflex regulation of RSNA. The primary reflex studied in this regard has been the cardiopulmonary afferent reflex. In essence, an increase in circulating blood volume using infusion of plasma or plasma expanders results in an increase in renal blood flow that is mediated by a decrease in SNA to the kidney. In this context, the PVN plays a critical role in mediating this inhibition of RSNA in a variety of species (Badoer et al., 2002; Li et al., 2003; Ramchandra et al., 2013). For example, microinjection of muscimol into the PVN caused attenuation of the inhibition of RSNA during volume expansion in conscious rabbits (Ng et al., 2004) and sheep (Ramchandra et al., 2013). Furthermore, lesions of the PVN in anesthetized rats attenuated the inhibition of RSNA and renal vasodilatation following volume expansion (Lovick et al., 1993; Haselton et al., 1994). Together these data indicate that in the normal state volume expansion stimulates a reflex that increases the level of GABAergic inhibitory input into the PVN and inhibits RSNA. In contrast, volume expansion during HF fails to activate central pathways (Akama et al., 1998) and results in an attenuated decrease in RSNA (Dibner-Dunlap and Thames, 1992; Patel et al., 1996b; Ramchandra et al., 2009b). Interestingly, this attenuated inhibition of RSNA during volume expansion is restored post exercise training (Pliquett et al., 2003; Zheng et al., 2006) probably mediated by an up-regulation of nNOS positive neurons within the PVN (Zheng et al., 2005).

It must be mentioned that in anesthetized animals, *dis*inhibition of the PVN attenuates the RSNA inhibition in contrast to conscious animals where inhibition of the PVN attenuated the RSNA inhibition. One important unanswered question is how inhibition of the PVN leads to an inhibition of RSNA. In this context, there are numerous inhibitory interneurons in the PVN. When muscimol is microinjected into the PVN, it is still unclear what subsets of neurons are being inhibited and whether inhibition of inhibitory interneurons occurs. This makes it hard to decipher neuronal networks within the PVN. Irrespective of these limitations, the PVN plays an important role in reflex regulation of RSNA and appears to be important in mediating the impaired reflex regulation of RSNA during HF.

## Conclusions

Understanding the role of RSNA in HF is difficult in humans where measurement is indirect at best. When considering findings from animal studies we must be mindful that the degree and model of HF, species and sex of the animals and presence of anesthesia all add complexity to the interpretation. Evidence does suggest that deterioration in cardiac function in HF is closely associated with elevations in RSNA, although it would appear that increased NE spillover only becomes significant once the ejection fraction is reduced below 30%. The exact mechanisms driving the increase in RSNA remain controversial, in part reflecting the diverse nature of the origins of HF. While any changes in sensitivity of the reflex regulation of SNA may simply be secondary to the increase in RSNA, there is emerging evidence that, at least in some instances, the peripheral chemoreceptors may be involved in driving the increase in RSNA. While in severe HF, there may be an impaired ability of the baroreflex to inhibit RSNA in males, it is difficult to suggest that the arterial baroreceptor reflex is the primary driver for the increase in RSNA. Additionally HF is characterized by impaired regulation of body water content. The PVN is highlighted as one central area which is associated with the impairment of the regulation of blood volume in HF, with an impaired ability of the PVN to inhibit RSNA in response to the increased blood volume observed in HF. Understanding the drivers for the increased RSNA and the variability that occurs between individuals is key to the successful management of HF.

### Conflict of interest statement

The authors declare that the research was conducted in the absence of any commercial or financial relationships that could be construed as a potential conflict of interest.
